# Communication difficulties in mechanically ventilated voiceless patients in intensive care units: A qualitative study

**DOI:** 10.1111/nicc.70037

**Published:** 2025-04-04

**Authors:** Sara Brambilla, Davide Ausili, Giulia Locatelli, Stefania Di Mauro, Giacomo Bellani, Michela Luciani

**Affiliations:** ^1^ Department of Medicine and Surgery University of Milano – Bicocca Monza Italy; ^2^ Centre for Medical Sciences – CISMed University of Trento Trento Italy; ^3^ Department of Anesthesia and Intensive Care Santa Chiara Regional Hospital, APSS Trento Trento Italy

**Keywords:** communication, intensive care units, mechanical ventilation, qualitative research, voicelessness

## Abstract

**Background:**

Mechanically ventilated patients are unable to verbally communicate due to the endotracheal tube or tracheostomy, rendering them temporarily ‘voiceless’. More and more patients are conscious during mechanical ventilation because of a new paradigm based on mild/no sedation. Communicating with conscious voiceless patients can be complex and frustrating, leading to negative outcomes and experiences for patients, family members and health care professionals.

**Aim:**

To explore the negative effects of the inability to communicate verbally among voiceless patients in intensive care units (ICUs), considering the perspectives of voiceless patients, health care professionals and family members.

**Study Design:**

This qualitative study uses Interpretive Description methodology. Semi‐structured interviews were conducted with patients, family members and health care professionals. Data were collected at three ICUs in Italy over 3 months. Data were analysed using the Rapid and Rigorous qualitative data analysis.

**Results:**

Forty‐three people were interviewed (10 patients, 13 caregivers, 13 nurses and 7 physicians). Three major themes were identified: perception of communication difficulties, negative impacts on relationships and emotions, and negative effects on care. These findings indicate that communication difficulties in ICU have negative emotional and psychological consequences for all participants and the health care provided.

**Conclusions:**

Effective communication with voiceless patients is essential for their well‐being and quality of care. Future research should focus on identifying and evaluating tailored communication methods for voiceless patients.

**Relevance to Clinical Practice:**

This study emphasizes the importance of interventions improving voiceless communication, including training health care professionals and critical care nurses in alternative communication strategies, providing psychological support to voiceless patients, and encouraging extended family presence.

**What is known about the topic**

- More and more patients are conscious during mechanical ventilation, although they are temporarily ‘voiceless’ because of the endotracheal tube or tracheostomy.
- Communicating with conscious voiceless patients can be complex and frustrating, leading to negative outcomes.

**What this paper adds**

- Communicating with conscious voiceless patients is associated with perceptions of communication difficulties, negative impacts on relationships and emotions, and negative effects on care, from patients, families and health care professionals' perspectives.
- Attention should be paid to designing and implementing interventions to improve voiceless communication, including training health care professionals and critical care nurses in alternative communication strategies, providing psychological support to voiceless patients, and encouraging extended family presence.

## 1 INTRODUCTION

Communication in the intensive care unit (ICU) has undergone significant transformations in the last two decades, marked by a shift from traditional practices of heavily sedating patients towards a new paradigm characterized by minimal sedation and regular patient awakening, which has been found to be associated with improved outcomes such as shorter time to extubation and lower tracheostomy rate in chronically ill mechanically ventilated adults.^1^ This led non‐sedated patients to receive mechanical ventilation. This way, they cannot speak due to factors such as intubation, ongoing treatment and illnesses,^2^ which render them temporarily voiceless.

Voicelessness ranks among the most distressing experiences for ICU patients, alongside thirst, breathing difficulties, and physical pain and discomfort.^3^ All voiceless patients in ICU face communication difficulties with different intensities: 28% encounter some difficulty, 23% experience moderate difficulty and 49% face extreme difficulty.^4^ Current communication practices, including communication aids like alphabet boards, are seldom successful and do not address the issue of voicelessness.^5^ Despite recognizing the communication challenges in the ICU, there is still no universally acceptable tool for voiceless patients to communicate.^6^ Voicelessness is exacerbated by patients' physical weakness, which hinders their ability to write or use alternative communication methods, and by the noise and disturbances of a high number of people and visitors in the ICU.^7^

Being voiceless generates negative emotions, leading to psychological distress encompassing anxiety, panic, frustration, anger and sleep disturbances.^8^, ^9^ Beyond its immediate effect on patients, voicelessness has a more extensive impact on both health care professionals (HCPs) and family members. HCPs struggle with the complexities of understanding and addressing the needs of voiceless patients, which, in turn, affects their daily responsibilities and job satisfaction.^10^ Family members experience significant frustration and a sense of helplessness when they cannot comprehend the patient's communication attempts.^11^

### 1.1 Background

The negative effects of voicelessness identified in previous studies highlighted the complexity of the phenomenon. Existing literature underscores the need to gain a deeper understanding of the impact of communication with voiceless patients, which could lead to the development of more efficient and alternative communication strategies. Research should encompass the perspectives of all those involved in the interaction to provide a better understanding of the complexity of communication.^12^ Research should also focus on exploring the experiences of family members involved in the communication process, not only patients and HCPs.^13^ However, as far as our knowledge extends, there are only a few studies in the literature that concurrently investigate the perspectives of all participants involved in communicating with ICU patients: one study explored communication challenges among patients, family members, and nurses and highlighted that, to an external observer, communication might seem smoother than it really is.^12^ The perception of nurses has often been investigated,^7^, ^14^, ^15^, ^16^, ^17^ but this does not apply to the perspective of physicians, which has often been disregarded.^10^, ^18^ Hence, qualitative studies are needed to explore patients' communication experience while awake and on an invasive mechanical ventilator, and to gain the perspectives of different health care professionals to comprehensively depict the phenomenon of communication in voiceless patients.^9^ Having this information will help uncover issues that may inform the development of more effective communication strategies and improve patient care in the ICU setting.

### 1.2 Objective of the study

Therefore, the objective of this study is to explore the negative effects resulting from the inability to communicate verbally among voiceless patients in ICU, considering the perspectives of voiceless patients, HCPs and family members.

## 2 DESIGN AND METHODS

This qualitative study uses Interpretive Description.^19^ This design was selected to generate in‐depth and contextually rich understandings of a complex phenomenon, with a particular emphasis on generating practical, applied knowledge that can inform health care practice.^19^ This study is reported in accordance with the Standards for Reporting Qualitative Research of the EQUATOR Network.

### 2.1 Setting and sample

This study's sample consisted of three subgroups: patients, family members and HCPs. We used purposeful sampling to recruit individuals (≥18 years old) who had previously been admitted to the ICU, had a minimum ICU stay duration of 48 h, had undergone mechanical ventilation either through a tracheostomy or an endotracheal tube, and had been discharged from ICU at least 48 h before the interview. Patients who receive mechanical ventilation without deep sedation often include those with conditions like pneumonia, where prolonged intubation and ventilation are required to support breathing. In such cases, deep sedation may not be necessary, allowing patients to remain conscious while still benefiting from ventilatory support. Regarding the use of analgesia, while there is no specific threshold defining ‘strong analgesia’ in this context, many awake patients receiving mechanical ventilation commonly receive low doses of fentanyl, either through IV or transdermal patches. This approach is used primarily to manage discomfort associated with the presence of the endotracheal tube. Patients who received such low‐dose fentanyl were included in the study, as this level of analgesia is generally compatible with consciousness and communication attempts. Exclusion criteria for patients were having psychiatric conditions or not remembering their ICU stay, as full recollection was necessary for meaningful participation in the study. After enrolling patients, we recruited their family members. Inclusion criteria for family members were being ≥18 years old and a family member of one of the study participants. The last subsample consisted of HCPs (nurses or physicians) working in the General, Neurosurgical or Cardiothoracic ICUs at a single Italian hospital. The inclusion criterion for HCPs was having been working in ICU for at least 6 months. Participants were contacted either through face‐to‐face interaction or via telephone communication and informed about the possibility of participating in the study.

Initially, we estimated a sample size of 45 of 60 participants divided as 15 of 20 patients, 15 of 20 family members, and 15 of 20 health care professionals. These estimates were made based on the phenomenon of interest in order to recruit a sample that could provide enough information power.^20^ In our case, in‐depth exploration of its underlying subjective experiential nature was needed. Thus, we looked for a sample size adequate to answer the research question to produce something worth documenting. Additionally, as specified by Thorne,^19^ the sample should be precisely crafted based on the knowledge that is needed, the options available for getting as close as possible to the phenomenon, and the most respectful approaches regarding ethical research guidelines.

### 2.2 Data collection tools and methods

Data were collected through semi‐structured interviews, one per participant, over a three‐month period. The interviews were conducted by a single research team member, an ICU nurse (SB), trained in qualitative interviewing and supervised by an expert nurse researcher in qualitative research (ML). Patients and family members were interviewed either through face‐to‐face or telephone interviews after the patient's discharge. For HCPs, face‐to‐face semi‐structured interviews were conducted. In the interviews, patients were asked to describe their methods of communication during their ICU stay, along with any associated feelings. Questions for patients focused on their ability to communicate needs, the methods and challenges they faced, what they wanted to convey to staff and family, and how communication difficulties affected their care. They were also asked to share examples of successful communication.

Similarly, family members and HCPs were asked about their interactions and communication strategies with their relatives or patients, with a particular emphasis on evaluating the effectiveness of these methods. Questions for family members explored their ability to communicate with the patient, how well they understood the patient's needs, and any challenges they faced. They were also asked to describe both successful and unsuccessful communication examples and reflect on how these interactions affected them and the health care team. Questions for health care professionals addressed how they interacted with conscious, mechanically ventilated patients, the types of patient requests they understood best, and their reactions to communication challenges. They were also asked to describe both successful and unsuccessful communication attempts, how communication barriers affected patient care, and the techniques they used to facilitate understanding. To enrich our understanding, all participants were encouraged to provide specific examples of their experiences. On average, the interviews lasted 30 min (16–72 min).

### 2.3 Data analysis

Interviews were audio‐recorded and anonymized. Then, they were analysed using the *Rapid and Rigorous qualitative data analysis*—RADaR.^21^ We chose the RADaR technique for its ability to provide a rapid yet systematic approach to qualitative data analysis. This technique allowed us to use straightforward tools, such as tables in word processing, to organize and progressively condense the data. Through its step‐by‐step data reduction process, RADaR enabled us to transform raw, textual data into a more manageable and structured format. This approach was particularly beneficial as it allowed us to analyse the data rigorously while accelerating the process by focusing on the most relevant segments in each phase. In summary, RADaR provided an efficient, practical method that facilitated both in‐depth and timely analysis of the interviews. Following this approach, two researchers (SB, ML) listened to the interviews multiple times and transcribed the sections relevant to the study question. The information considered most significant was extracted and organized within tables by two researchers. A review process of raw data contained in the tables was undertaken and only the relevant information aligning with the study's objective was kept. This process reduced data volume, resulting in progressively shorter and more concise tables, where essential data were arranged in a precise and schematic manner. Two researchers (SB, ML) identified the main themes that provided an explanatory framework for the phenomenon of interest. Then, the entire research team reviewed this framework of findings, where also relevant quotes were incorporated. The process of interpretation involved a dynamic discussion on the thematic structure of the findings. The interviews were initially analysed separately for each group (patients, family members and health care professionals) to avoid cross‐influence and capture each group's unique perspective. Subsequently, the analyses were combined to identify commonalities and differences across the groups, allowing us to highlight both shared themes and distinct experiences related to communication.

### 2.4 Rigour

To ensure integrity, we clearly outlined the research process to clarify the research question we started from, the definition of data sources, and the ultimate interpretation of the findings.^22^ To ensure credibility, we triangulated data sources by exploring patients', family members' and health care professionals' perspectives on the phenomenon of interest.^19^ To ensure validity and reliability, data analysis was team‐based, with peer debriefing and critical discussion among the team members.^23^ The principles of coherence and credibility guided our methodological choices as we aimed to produce a detailed, in‐depth and meaningful report of the phenomenon of interest.^19^, ^22^, ^23^

## 3 RESULTS

A total of 43 participants were interviewed: 10 patients, 13 family members and 20 HCPs. Most patients were males, with a mean age of 59.9 years. Most family members were females and visited their relatives in the ICU in person. Among HCPs, there were 13 nurses and 7 physicians, with a mean age of 29.7 years (Table 1).

**TABLE 1:** Socio‐demographic characteristics of the sample.

Sample	Characteristics	*N* (%)
Patients (*n* = 10)	Gender	
	Female	4 (40%)
	Male	6 (60%)
	Mean age in years	59.9 [CI 25–78]
	Mean length of stay in ICU in days	23.9 [CI 4–50]
Family members (*n* = 13)	Gender	
	Female	9 (69%)
	Male	4 (31%)
	Family members who visited their relative in person	9 (69%)
	Family members who video‐called their relative	4 (31%)
Health care professionals (*n* = 20)	Gender	
	Female	15 (75%)
	Male	5 (25%)
	Mean age in years	29.7
	Profession	
	Nurse	13 (65%)
	Physicians	7 (35%)

*Note*: Data are presented as number (%), except for age and length of stay that are presented as number [CI].

Abbreviations: CI, confidence interval; ICU, intensive care unit.

**FIGURE 1:**
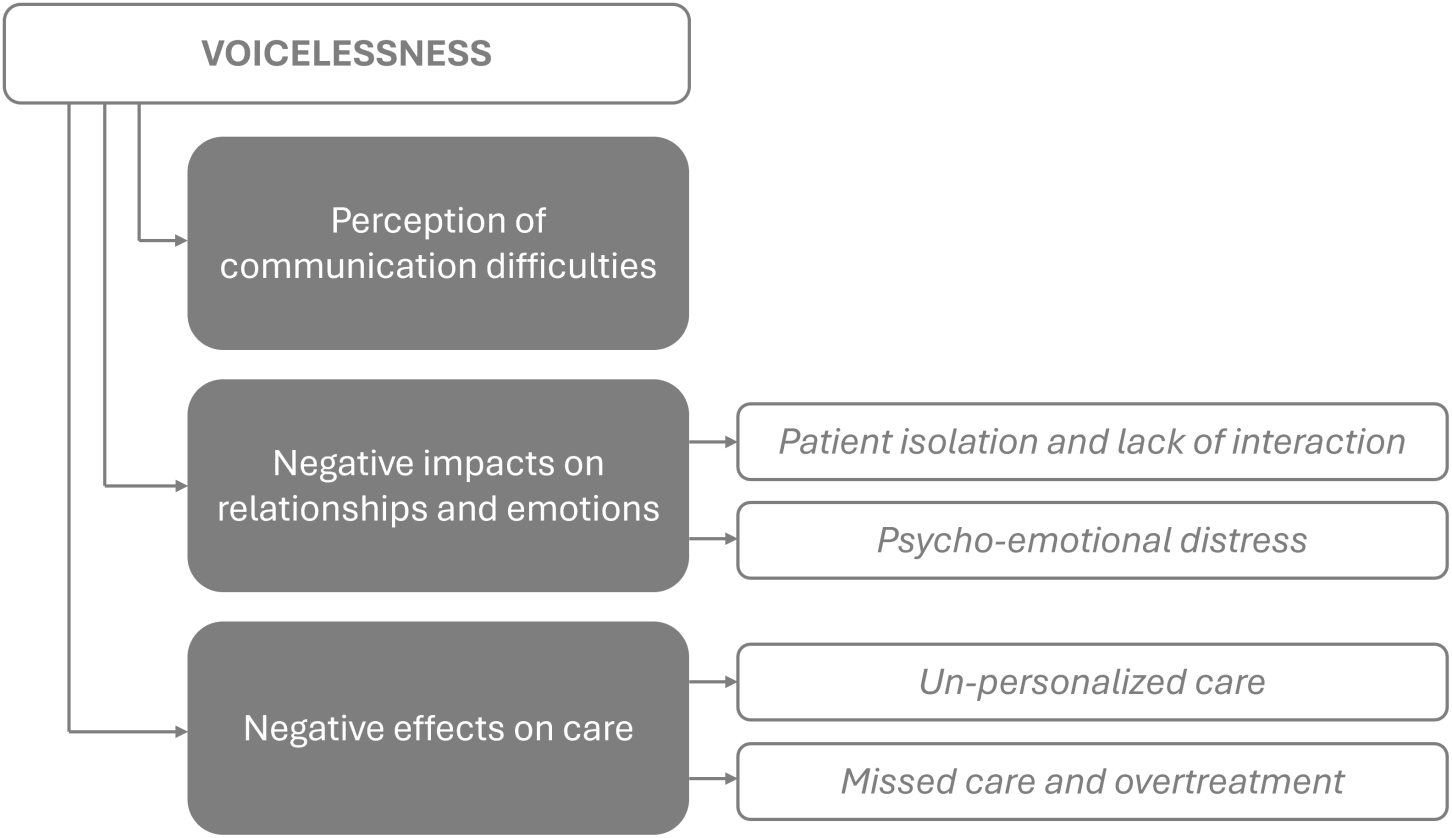
Graphical representation of themes.

Three major themes were identified. The first theme was the perception of communication difficulties, which is how participants perceived and experienced voicelessness. The second theme was the negative impacts on relationships and emotions, which delves into the repercussions of voicelessness, encompassing aspects of patient isolation and limited interaction, and psychological and emotional distress experienced by patients and their families due to these circumstances. The last theme was the negative effects of voicelessness on care, including issues such as unpersonalized care and missed care and overtreatment (Figure 1).

### 3.1 Perception of communication difficulties

Despite variations in the extent and kinds of challenges associated with voicelessness among patients, family members and HCPs, all participants described it as a relevant issue. Family members tended to focus more on their relatives' health outcomes and well‐being rather than the voicelessness challenges encountered (Table 2, Q1, Q2). Patients reported that communication and interaction were fundamental to them, and they claimed to have repeatedly tried to communicate. The range of needs they attempted to communicate extended beyond just physiological aspects (Table 2, Q3), as a nurse stated:

At 11 p.m., a colleague called me because he was having trouble understanding his patient. We addressed him, saying, ‘We are having difficulty understanding you. Please, help us understand if it's related to your positioning or something else.’ Eventually, I managed to lip‐read and asked, ‘Sugar?’ realizing the patient was disoriented. He confirmed by mouthing, ‘Yes, sugar’. As it turned out, he actually wanted us to play songs by the Italian singer Zucchero [Authors' note: Sugar in Italian is “zucchero” which is also the name of an Italian singer]. Three of us spent approximately 40 minutes attempting various methods—offering water, inquiring about family, adjusting his position—only to discover his request was listening to music played by Zucchero. It took us a long time to figure it out, but he never gave up. (NU07)

**TABLE 2:** Themes and selected participants' quotes.

Theme	Participant's quote
Perception of communication difficulty	**Q1**: Personally, I don't see any repercussions from this [the difficulty in communication], but I'm the son of a patient who is currently recovered and has not experienced any psychological consequences. (FM02) **Q2**: You were all amazing. You were present. I have never seen you irritated. And I know that my daughter has good memories of you. (FM03) **Q3**: My hospitalization was sudden, and I had left many things pending, some of which were quite urgent. So, there was also the need of asking my family to take care of things at home, like ‘turn off the heaters’. (PT08) **Q4**: We often forget the psychological aspects. (NU01) **Q5**: If you don't understand what the patients want, you can even say ‘the parameters are fine’ but it's just a superficial thing. (NU20) **Q6**: It was distressing because I wasn't in a condition to express myself. I felt a bit angry because it seemed absurd to me that [the doctors and nurses] couldn't come up with a way to communicate. (PT05) **Q7**: I experienced a sensation I had never felt before, the feeling of being unable to do anything for him. Even though you're in the hospital, there's not much you can do. In those moments, we felt powerless. (FM01) **Q8**: I often don't feel good enough. I feel like running away because I can't solve it. The burden of not being able to help is suffocating. (NU07)
Negative impacts on relationships and emotions *Patient isolation and lack of interaction*	**Q9**: Often, I said one thing, they understood something else, and I, frustrated, gave up, keeping my discomfort to myself. (PT06) **Q10**: It is easier to say, ‘Look, we'll try later because I have to administer the therapy now,’ to put it to an end. (NU18) **Q11**: The video call was brief because we couldn't talk. I was the only one speaking. There was a real barrier between us and her. (FM12)
Negative impacts on relationships and emotions *Psycho‐emotional distress*	**Q12**: I carry memories with me, I have this feeling of psychological mistreatment, let me explain: I asked for water, they said they couldn't give it to me, and I didn't understand. (PT09) **Q13**: I couldn't speak, and I thought I had lost my voice. I thought I had lost my voice because of the accident, but it turned out to be because I was intubated. This thing in my head drove me crazy; it felt like I was hearing voices. (PT10) **Q14**: We often forget the psychological perspective. And it increases the patient stress, and stress affects the whole system. (NU01) **Q15**: It was distressing because I had a long period of coma, and when I woke up, I couldn't see my family, so I couldn't see my husband. I even started to imagine that my husband had died, even though he wasn't in the accident. It was distressing because I wasn't in a condition to express myself. I was afraid he was no longer there, but I didn't know how to tell him. That phase was a bad experience. I felt a bit angry because it seemed absurd that they couldn't come up with a way to communicate. I didn't have fine motor skills, and my handwriting was illegible. (PT05) **Q16**: Gradually, patients isolate themselves: as they struggle to make themselves understood, they shut down and somehow avoid interacting and calling for you. (NU07)
Negative effects on care *Un‐personalized care*	**Q17**: Sometimes it was clear that he wanted to say things, but we understood something different. (FM02) **Q18**: Perhaps it's a very simple thing, a very simple request but it is difficult to fulfil because understanding is challenging. (NU15) **Q19**: In my opinion, we provide care, I mean, the technical quality may not be lacking, but in the end, we don't get involved and interact with the patient, and that's necessary! If you put yourself on the other side of the bed, you feel excluded, you feel treated more like a thing than a person. I can still prepare the norepinephrine, but maybe you're telling me that your foot hurts and I didn't notice. For someone in the bed who isn't thinking about anything else that matters. That's what creates quality; it all depends on perspectives: I still prepared the norepinephrine for you, but, if I didn't realize your little finger hurt, it means I didn't care for you properly anyway. (NU18) **Q20**: It's not personalized care: we see the clinical alterations, but we can't understand the patient's real needs. (PH14)
Negative effects on care *Missed care and overtreatment*	**Q21**: Many times, trying to interpret and experimenting, the risk is to do useless things. (PH17) **Q22**: [The difficulty of communicating] greatly influences [care] because even something as simple as pain is important and affects vital signs. So, effectively communicating with the patient is important. Sometimes you can treat hypertension with antihypertensive drugs, when maybe a pain reliever would be enough if it's caused by the pain. (NU03) **Q23**: You can't understand their needs. Sometimes patients get so agitated, and you have to take some kind of action with medications or restraints. (NU10) **Q24**: You can't do your job. If you can't understand your patient's needs, you can't implement strategies to help them and everything that can be done to support them. (PH17)

Abbreviations: FM, family member; NU, nurse; PH, physician; PT, patient; Q, quote.

HCPs, on the other hand, primarily focus on the patient's clinical needs (Table 2, Q4). Nevertheless, the interviews highlighted the importance the HCPs placed on identifying patients' needs correctly through effective communication (Table 2, Q5). All participants reported experiencing negative emotions when they were unable to communicate or understand each other. Patients mainly reported anger, irritation, and frustration (Table 2, Q6). Family members mentioned a sense of helplessness and sadness (Table 2, Q7). HCPs reported feeling inadequate, uncomfortable, upset and frustrated (Table 2, Q8).

### 3.2 Negative impacts on relationships and emotions

The second theme referred to the negative impacts of communication difficulties and voicelessness on relationships and emotions. This theme delves into the repercussions of voicelessness, encompassing aspects of patient isolation and limited interaction, and psychological and emotional distress experienced by patients and their families due to these circumstances.

#### 3.2.1 Patient isolation and lack of interaction

Participants stated that during the first days of hospitalization, communication challenges induced them to seek alternative communication methods, such as lip‐reading, gestures or alpha‐numeric boards. After several unsuccessful attempts, patients, family members and HCPs reported becoming fatigued and angry, and stopped trying to communicate (Table 2, Q9–Q11). The distancing and the negative emotions experienced due to repeated failures in communication and the lack of understanding led patients to isolate themselves, feeling alone. Without social interaction, participants felt estranged from reality, as a patient reported: ‘*Not being able to speak excluded me from reality. I no longer knew what day it was, I lost track of time. I didn't know what my children were doing, how my grandchildren were*’ (PT06).

#### 3.2.2 Psycho‐emotional distress

The absence of communication and social interaction affected the psychological and emotional state of patients, who are already in a vulnerable condition. Many patients described the discomfort they experienced in the ICU (Table 2, Q12, Q13). Patients have found themselves in a stressful situation, negatively impacting their well‐being and humour (Table 2, Q14). Some individuals reported a difficult‐to‐explain feeling of ‘something missing’ as if they were lost without the ability to communicate (Table 2, Q15). HCPs also perceived the negative influence of communication barriers on patients (Table 2, Q16). These negative feelings sometimes persisted in patients even after the discharge, indicating the presence of post‐traumatic symptoms, as a patient stated: ‘*Even today, I still have nightmares at night about not being able to breathe and not being able to tell anyone*’ (PT06). Both family members and HCPs confirmed the deterioration of the mood and emotional state of the individuals due to social isolation and the absence of effective interactions: ‘*After the frustration, day after day, patients gradually close off, shut down, isolate themselves, and no longer interact*’ (NU08).

### 3.3 Negative effects on care

The third theme referred to the negative effects of voicelessness and the consequent communication difficulties on care, especially the resulting un‐personalized care, missed care and overtreatment as perceived by participants.

#### 3.3.1 Un‐personalized care

HCPs reported that communication allows them to identify patients' needs, but with voiceless patients, it became challenging. This often led to misunderstandings (Table 2, Q17, Q18). HCPs stated that misinterpretations increased the risk of errors in identifying the needs of the patients. This was confirmed by a patient:

I had a small tube in my nose, and that morning they had put a new bandage on it, but it was bothering me. It was itchy, and I didn't know how to say it. I couldn't touch my nose, so I shook my head. I got agitated, they kept telling me to stay still and calm, but no one understood. They must have thought I was crazy. (PT06)

HCPs described that the inability to comprehend the true needs of patients may lead to standardized and detached care, resulting in it being inadequate (Table 2, Q19). On the contrary, they described effective communication as essential to understand patients' worries and problems, and find the underlying causes of clinical alterations and patient agitation (Table 2, Q20).

#### 3.3.2 Missed care and overtreatment

HCPs stated that the lack of communication with voiceless patients increases the likelihood of providing inadequate care, because the patients might be misunderstood (Table 2, Q21, Q22). It was underlined that this is particularly true for agitated patients who are challenging to understand (Table 2, Q23, Q24). In fact, HCPs emphasized the difficulties encountered in their job when interacting with voiceless patients, and how crucial it is to fully understand patients' requests to meet their needs and offer the best care possible. A patient described a situation of missed care, where their need was not addressed: ‘*It was a situation of great frustration and discomfort because it was clear that something was hurting me, and I couldn't communicate how they should have moved and readjusted me, and the discomfort persisted*’ (PT08). A situation of overtreatment was explained by a nurse: ‘*You can't understand their needs. Sometimes patients get so agitated, and you have to take action with medications or restraints*’ (NU10).

## 4 DISCUSSION

This study investigated the negative effects of the inability to verbally communicate among non‐sedated, mechanically ventilated patients in the ICU, through the perspectives of voiceless patients, health care professionals and family members. We found that all participants experienced communication difficulties, although at varying degrees, and that such difficulties had negative consequences in all three groups. The main insight provided by this study is that the lack of communication due to voicelessness has a negative impact on health care, resulting in missed care or overtreatment. This is relevant because it highlights the opportunity for developing interventions aimed at mitigating communication barriers associated with voicelessness. Implementing such interventions has the potential to enhance the quality of care provided while reducing situations of missed care or overtreatment.

In this study, HCPs underlined the essential role of communication in providing personalized care and correctly identifying patients' needs. They claimed that, without understanding patients' requests, it is impossible to provide adequate care. HCPs also affirmed that voicelessness‐related misunderstandings have negative consequences for their work and patient care, with the overuse of medications on one hand and situations of missed care on the other. Our findings are consistent with previous literature, which has emphasized that a lack of communication leads to misunderstandings.^14^ Often, these misunderstandings impact patient care, making it challenging to treat patients.^15^ In previous studies, more than 75% of the staff reported that communication issues interfered with the provision of adequate care, psychological and emotional aspects, and the assessment of patient symptoms.^10^, ^24^ On the other hand, effective communication has been proven to enhance the patient's recovery and potentially reduce their time in the ICU, because it helps them cope with their situation, improving their outcomes and well‐being.^25^, ^26^

Some HCPs also reported that the most challenging cases to manage are patients with psychomotor agitation. This is primarily related to the increased complexity in interpreting these patients' needs and the management of medical devices. Consistent with our findings, a study described the potential negative consequences of agitation in patients in a critical care context, such as device repositioning or increased oxygen consumption, which often require interventions by health care professionals, such as sedation or the use of anxiolytics and analgesics.^27^ Lastly, in our study, HCPs reported emotions of helplessness, inadequacy and frustration when interacting with voiceless patients. HCPs expressed the same feelings in other studies: feelings of defeat, helplessness and inadequacy relating to their role,^14^ and anger and frustration.^10^, ^14^, ^15^, ^16^, ^17^, ^18^ This finding is crucial because effective communication is vital for HCPs, as prior research has indicated that poor communication can lead to adverse outcomes, including burnout, job‐related stress and increased employee turnover.^28^

To the best of our knowledge, this study is among the first to investigate the perspective of family members, and it is among the few that consider the perspectives of both nurses and physicians, which is relatively uncommon in the existing literature. A previous study provided an important description of the phenomenon of communication impairments in voiceless patients by exploring the perspectives of patients, families and nurses.^12^ We built upon that knowledge by also including the perspectives of other health care professionals who still play a role in the phenomenon and by doubling the sample size to ensure a thick description and increase the internal validity and reliability of our findings. Our results show that family members approach the situation and interact with their relatives in two opposite ways. A portion of family members reported experiencing positive emotions due to the opportunity to connect with their loved ones, despite them being voiceless. On the contrary, the other portion declared experiencing negative emotions due to communication difficulties and numerous misunderstandings. Similar results were highlighted in previous literature where family members often showed ambivalent attitudes,^29^ with some of them feeling such intense negative sensations that they wished for greater sedation for the relative to no longer feel helpless.^11^ However, in our study, a subset of patients also reported that they found it easier to communicate with their family members because they seemed to grasp things more quickly and effectively than HCPs. Participants identified their relatives as a resource: they assisted in improving communication and understanding, supported the patient, and provided emotional support, transmitting familiarity, strength and courage. These results align with previous studies in which patients stated that family members are better than HCPs at interpreting what they wanted to express^30^ and that they would have liked their family members by their side to receive their emotional support.^31^ In fact, longer visits and the presence of a relative are considered protective factors for patients because they increase patients' connection with the ‘real’ world.^32^ Our research suggests that promoting family presence in ICU settings could be crucial in improving communication with voiceless patients.

We found that patients experience negative emotions due to voicelessness, such as anxiety, anger, frustration and helplessness, which are consistent with those described in other studies.^7^, ^30^, ^33^, ^34^, ^35^, ^36^, ^37^ These negative effects still affected patients even months after discharge from the hospital, resulting in symptoms of post‐traumatic stress, such as nightmares and unpleasant memories. This is coherent with previous studies, where authors reported that being hospitalized in an ICU can readily lead to psychological issues, negative emotions and post‐traumatic stress disorders for the majority of patients.^9^, ^13^, ^38^, ^39^ Our findings could further support the role of communication difficulties in shaping post‐intensive care syndrome. By highlighting the negative emotions and psychological distress experienced by patients and the negative effects on the care provided due to voicelessness, our study underscores the need for improved communication strategies.

### 4.1 Limitations

This study has some limitations. Firstly, our sample was recruited from a single hospital. Secondly, we conducted the study during the COVID‐19 pandemic, when visits from family members were partially restricted (e.g., limited visiting hours and mandatory face masks). However, a strength of this study is that our sample is composed of participants from three different intensive care wards with diverse characteristics in terms of age, gender, average length of stay in the ICU for patients, or profession and experience for HCPs, ensuring a high information power of this sample for a meaningful clinical understanding.

### 4.2 Implications and recommendations for practice

Future research should focus on testing new communication strategies tailored for voiceless patients in intensive care settings, as well as exploring if the presence of family members could help patients cope with voicelessness. The results of this study could be particularly useful to health care professionals and critical care nurses as they highlight the need for implementing strategies to enhance and improve communication between HCPs, voiceless patients and their families. Based on that, the necessity to train HCPs and critical care nurses in alternative communication strategies stands out.

## 5 CONCLUSION

We conducted a qualitative study to investigate the negative effects of the inability to verbally communicate among voiceless patients in the ICU, considering the perspectives of patients, HCPs and family members. We found that voicelessness has negative effects on the well‐being of patients, job satisfaction of HCPs, and the health care provided in the ICU, with missed care or overtreatment linked to the inability to comprehend patients.

Our findings highlight the need for interventions aimed at enhancing communication between HCPs, voiceless patients and their families, contributing to improving patient well‐being and potentially reducing their time in intensive care settings. Based on our results, it is imperative to train HCPs in alternative communication strategies to engage effectively with voiceless patients. Moreover, providing psychological support for both patients and their family members throughout the recovery is essential to help them cope with these challenges. Lastly, the presence of family members should be encouraged beyond regular visiting hours to support the patients.

## CONFLICT OF INTEREST STATEMENT

Prof. Giacomo Bellani declares equities in DICO Technologies SRL. All the other authors declare no conflict of interest.

## ETHICS STATEMENT

Ethical approval was obtained from the Hospital's Ethical Committee (Protocol Number: TICOM2021, 30 December 2021).

## PATIENT CONSENT STATEMENT

Before beginning study procedures, participants signed an informed consent form, a data privacy agreement and consented to the audio‐recording of the interview.

## Data Availability

Data on which the results of this study are based are already presented in the manuscript. Additional data may be requested to the corresponding author.
